# Nasal glomus tumor: A rare nasal tumor with diffuse and strongly positive synaptophysin expression

**DOI:** 10.1111/pin.12866

**Published:** 2019-11-04

**Authors:** Shiori Meguro, Yukiko Kusama, Sayomi Matsushima, Haruna Yagi, Hideya Kawasaki, Isao Kosugi, Takashi Tsuchida, Satoshi Baba, Yasunori Enomoto, Seiji Hosokawa, Toshihide Iwashita

**Affiliations:** ^1^ Department of Regenerative and Infectious Pathology Hamamatsu University School of Medicine Shizuoka Japan; ^2^ Division of Diagnostic Pathology Nagano Municipal Hospital Nagano Japan; ^3^ Department of Diagnostic Pathology Hamamatsu University Hospital Shizuoka Japan; ^4^ Department of Otorhinolaryngology Hamamatsu University Hospital Shizuoka Japan

*To the Editor*

Herein, we report a case of a diffusely and strongly synaptophysin‐positive nasal glomus tumor that required differential diagnosis from other tumors with endocrine features. Glomus tumors arise from the glomus body, a specialized form of arteriovenous anastomosis that regulates the temperature in the distal extremities. Glomus tumors very rarely occur in visceral organs, where glomus bodies are supposed to be absent, and many of these rare cases occur in the digestive organs, particularly the stomach. Glomus tumors occurring in the extremities do not express synaptophysin, an excellent diagnostic marker for neuroendocrine differentiation. However, approximately 10% of gastric glomus tumors focally express synaptophysin.^1^ To date, there have been no reports of diffuse and strong synaptophysin expression in glomus tumors.

A 25‐year‐old Japanese woman had occasionally recognized spontaneous bleeding from the right nasal cavity. However, she presented herself to a hospital on account of persistent epistaxis. Afterwards, the patient was referred to the Department of Otorhinolaryngology at our hospital for medical evaluation and appropriate treatment. Anterior rhinoscopical examination revealed the presence of the reddish and hemorrhagic mass in the right middle nasal meatus. Contrast‐enhanced computed tomography (CT) demonstrated that the mass (32 × 31 × 15 mm) occupying the right nasal cavity showed no apparent bony destruction (Fig. 1a). The radiological findings indicated a tumor with hypervascularity localized in the right nasal cavity. After the patient underwent nasal polypectomy, she was discharged immediately without any complications. The patient was regularly observed by the otorhinolaryngologists for1 year after surgery, but no recurrence was detected.

**Figure 1:**
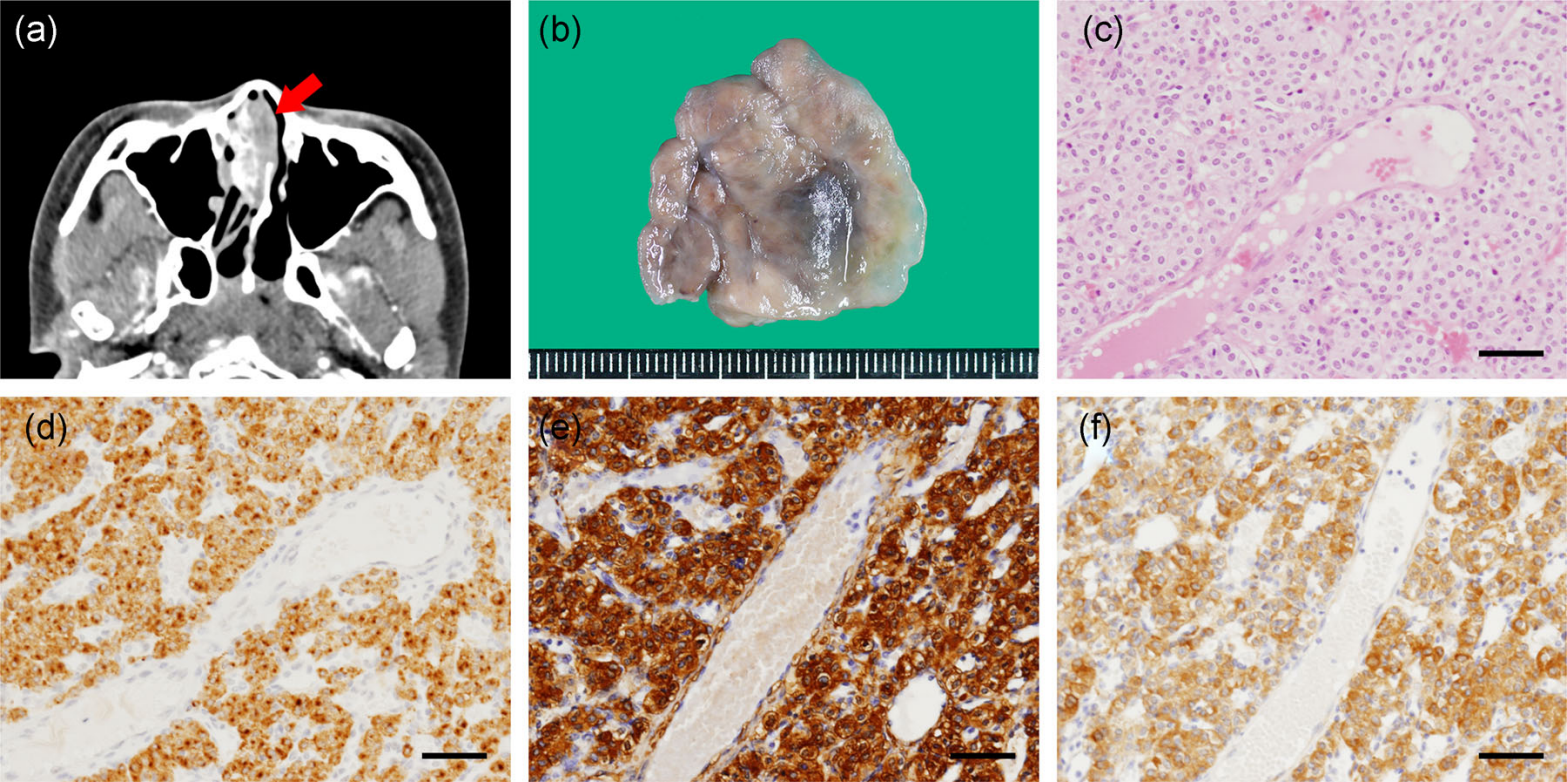
Macro‐, and microscopic evaluation of the resected specimen. (**a**) A contrast‐enhanced computed tomography image of the intranasal tumor. The arrow indicates the hypervascular lesion occupying the right nasal cavity. (**b**) A gross view of the excised tumor. (**c**) Hematoxylin and eosin staining (×200). Round‐to‐oval‐shaped tumor cells were present around small vessels. The tumor cells showed positive expression for (**d**) synaptophysin (×200), (**e**) αSMA (×200), and (**f**) MYO1B (×200). Scale bars = 50 µm (**c–f**).

A macroscopic view of the resected specimen revealed a solid tumor measuring 30 × 30 × 8 mm (Fig. 1b). Microscopic examination revealed that the tumor exhibited a perivascular growth pattern. The tumor consisted of numerous blood vessels of varying sizes from capillary to venule, and monomorphic cells with round‐to‐oval nuclei and a pale cytoplasm (Fig. 1c). Tumor cells showed no apparent cytological atypia. The cell membranes were well defined and mitosis was almost undetectable. No necrotic tissue was identified in the tumor. No tumor invasion into surrounding normal tissues was observed.

Immunohistochemical staining showed that the tumor was diffusely positive for synaptophysin (Fig. 1d), α‐smooth muscle actin (Fig. 1e), and myosin 1B (MYO1B; a pericyte marker)^2^ (Fig. 1f). Most parts of the tumor tested negative for high‐molecular weight caldesmon (hCD; a marker for smooth muscle cells) (Fig. S1a). The Ki‐67 proliferative index was lower than 3% (Fig. S1b). The tumor cells tested negative for melan‐A (Fig. S1c), HMB45 (Fig. S1d), chromogranin A (Fig. S1e), CD56 (Fig. S1f), insulinoma‐associated protein 1 (Fig. S1g), β‐catenin (Fig. S1h), AE1/AE3 (Fig. S1i), and S100 (Fig. S1j). Antibodies used in this study are detailed in Table S1.

To make a definitive diagnosis, it was necessary to distinguish the present tumor from other sinonasal tumors that would also present a perivascular growth pattern, such as perivascular epithelioid cell tumor (PEComa), sinonasal glomangiopericytoma (SN‐GPC), paraganglioma, and neuroendocrine tumors. Since the present tumor tested negative for melan‐A, HMB45, β‐catenin, CD56, chromogranin A, and S100, we could rule out PEComa, SN‐GPC, paraganglioma, and neuroendocrine tumors. Based on these findings, the tumor was histologically and immunohistochemically diagnosed as a glomus tumor with diffuse and strong synaptophysin expression. To the best of our knowledge, fewer than 40 cases of sinonasal tract glomus tumor have been reported in the English language literature. Furthermore, to our knowledge, no prior studies have described sinonasal tract glomus tumors expressing synaptophysin and other neuroendocrine markers.

The most interesting biological features of the present glomus tumor were the diffusely and strongly expressed synaptophysin regardless of the lack of neuroendocrine differentiation. In general, glomus tumors in peripheral soft tissues do not express synaptophysin. However, 11 cases of glomus tumor arising from various visceral organs (the stomach (n = 5), esophagus (n = 2), duodenum (n = 1), bronchus (n = 1), kidney (n = 1), and liver (n = 1)) have been reported to express synaptophysin in the English language literature.^1^, ^3^, ^4^ In those reports, all glomus tumor cases tested focally positive for synaptophysin and negative for CD56 and chromogranin. Most cases of glomus tumors positive for synaptophysin exhibit either histological atypia or clinically malignant behavior,^4^ whereas the glomus tumor in the present case was benign despite being diffusely and strongly positive for synaptophysin. From these results, the expression of synaptophysin in the present nasal glomus tumor does not seem to be related to malignant characteristics.

Synaptophysin is involved in synaptic transmission, the kinetics of synaptic vesicle endocytosis in neurons, and in the pathway by which microvesicles are recycled in both nonneuroendocrine and neuroendocrine cells. Pinocytotic microvesicles are found during pinocytosis, a form of endocytosis that is characteristic of pericytes. We hypothesize that the diffuse and strong synaptophysin expression in the present glomus tumor may be associated with the increased rate of formation of pinocytic vesicles in the tumor cells. However, tumor distortion due to formalin fixation made it difficult to confirm the existence of pinocytic vesicles in the cytoplasm of the glomus tumor cells via electron microscopy. One study successfully conducted an electron microscopic investigation on a case of a visceral glomus tumor with synaptophysin expression and found pinocytic vesicles, which were clearly visible in a gastric glomus tumor.^5^ However, since it was not compared with the number of pinocytic vesicles in synaptophysin‐negative tumor cells of the same glomus tumor, it was unclear whether the amount of pinocytic vesicles actually increased in synaptophysin‐positive tumor cells.

In this report, we have presented an extremely rare case of a nasal glomus tumor with diffuse and strong synaptophysin expression. To the best of our knowledge, this is the first case report of a glomus tumor that tested strongly and diffusely positive for synaptophysin in the English language literature. Further analysis of similar rare cases will be necessary to elucidate the function of synaptophysin in glomus tumors.

## DISCLOSURE STATEMENT

None declared.

## AUTHOR CONTRIBUTIONS

Concept and design: SM and TI; data analysis: YK, SM, HY, HK, and IK; immunohistochemical analysis: TT, SB, and YE; preparation of manuscript and figures: SM and TI. All authors have read and approved the manuscript.

## Supporting information

Additional Supporting Information may be found in the online version of this article at the publisher's website.

Figure S1. Negative immunohistochemical staining was evident for (**A**) hCD (×200), (**B**) Ki‐67 (×200), (**C**) melan‐A (×200), (**D**) HMB45 (×200), (**E**) chromogranin A (×200), (**F**) CD56 (×200), (**G**) insulinoma‐associated protein 1 (INSM1) (×200), (**H**) β‐catenin (×200), (**I**) cytokeratin AE1/AE3 (×200), and (**J**) S100. Scale bars = 50 µm

Table S1. Antibodies used in this study and the methods of antigen retrieval
