# Supplementary material for: Nasal glomus tumor: A rare nasal tumor with diffuse and strongly positive synaptophysin expression
Source: Pathol Int. 2019 Nov 4;69(11):672–4. doi: 10.1111/pin.12866 (PMC6899972; doi:10.1111/pin.12866)
Supplement: Supplementary file 1 — Figure S1. Negative immunohistochemical staining was evident for (A) hCD (×200), (B) Ki‐67 (×200), (C) melan‐A (×200), (D) HMB45 (×200), (E) chromogranin A (×200), (F) CD56 (×200), (G) insulinoma‐associated protein 1 (INSM1) (×200), (H) β‐catenin (×200), (I) cytokeratin AE1/AE3 (×200), and (J) S100. Scale bars = 50 µm [file PIN-69-672-s001.docx]

**Supporting Figure 1**


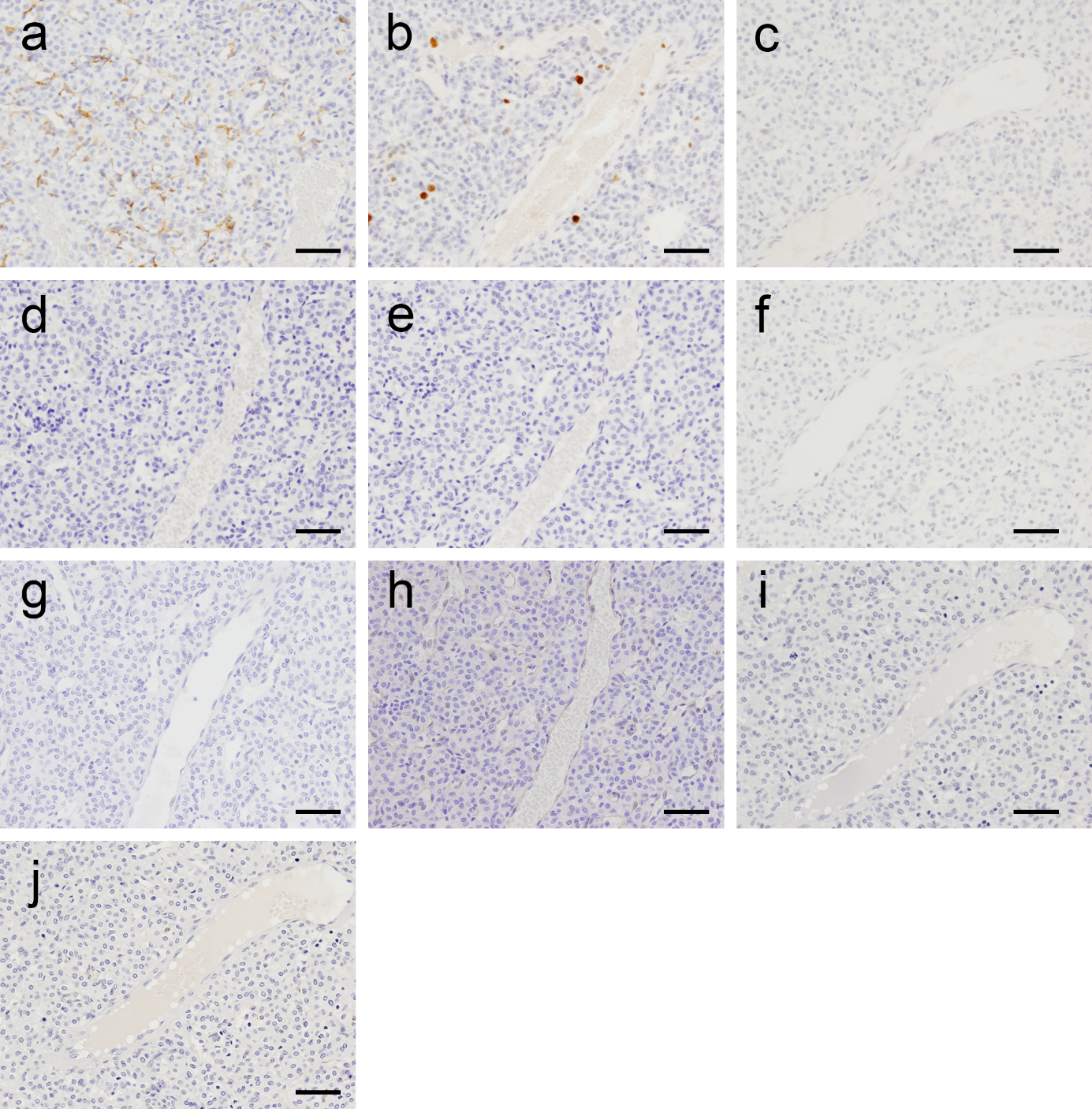


Supporting Figure 1: Negative immunohistochemical staining was evident for (a) hCD (×200), (b) Ki-67 (×200), (c) melan-A (×200), (d) HMB45 (×200), (e) chromogranin A (×200), (f) CD56 (×200), (g) insulinoma-associated protein 1 (INSM1) (×200), (h) β-catenin (×200), (i) cytokeratin AE1/AE3 (×200), and (j) S100. Scale bars = 50 µm.
